# First person – Jelmer Hoeksma

**DOI:** 10.1242/dmm.047282

**Published:** 2020-09-24

## Abstract

First Person is a series of interviews with the first authors of a selection of papers published in Disease Models & Mechanisms, helping early-career researchers promote themselves alongside their papers. Jelmer Hoeksma is first author on ‘Cercosporamide inhibits bone morphogenetic protein receptor type I kinase activity in zebrafish’, published in DMM. Jelmer is a PhD student/technician in the lab of Jeroen den Hertog at Hubrecht Institute, Utrecht, The Netherlands, identifying biologically active fungal compounds and uncovering their mode of action *in vivo*.


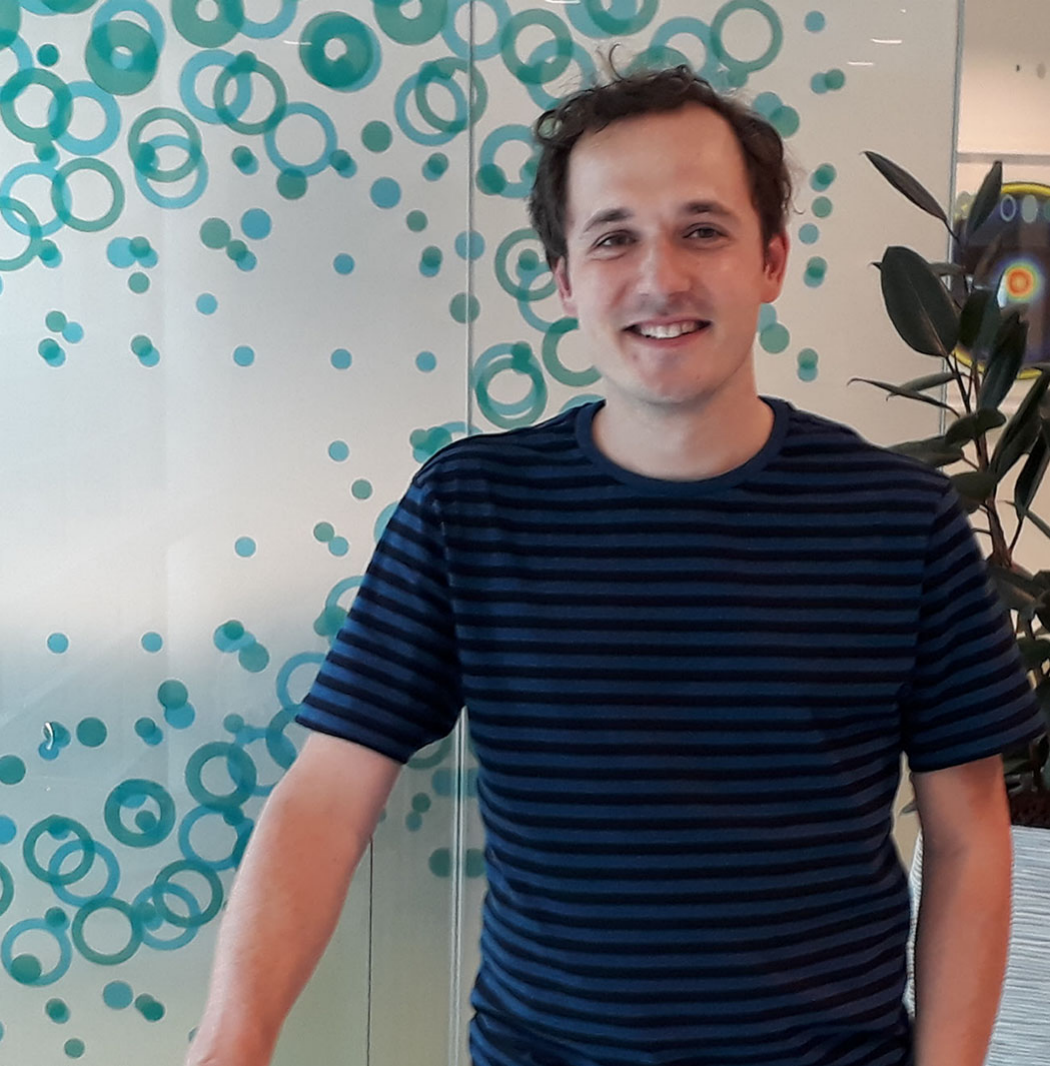


**Jelmer Hoeksma**

**How would you explain the main findings of your paper to non-scientific family and friends?**

We are trying to find (new) compounds from fungi, which hopefully can be used for therapeutic purposes. To do this, we grow the fungi on liquid medium, in which they secrete all sorts of compounds. We filtrate the medium and then test it on zebrafish eggs and look for abnormalities in development, which give clues about the way these compounds work. We tested over 10,000 fungi this way.

One particular fungus showed the same effect in zebrafish embryos as established inhibitors of a specific group of proteins called BMPs. Overactive BMPs are involved in several diseases such as fibrodysplasia ossificans progressiva. People suffering from this disease have their muscle tissue progressively replaced with bone tissue. So, this fungus might produce a compound to counteract this process. We purified and identified a compound called cercosporamide from the fungal filtrate. Next, we tested cercosporamide alongside and in combination with known BMP inhibitors in human cells and zebrafish cells and established that cercosporamide is indeed a BMP inhibitor.

**What are the potential implications of these results for your field of research?**

It opens up the potential use of cercosporamide as a BMP inhibitor. Furthermore, cercosporamide is structurally a completely different molecule from most commonly used BMP inhibitors. Thus, even if cercosporamide itself turns out to be unusable as a therapeutic drug, a completely different class of structurally related chemicals can now be investigated as BMP inhibitors.

**What are the main advantages and drawbacks of the model system you have used as it relates to the disease you are investigating?**

Zebrafish embryos develop rapidly, are transparent and the eggs are laid in large quantities. Furthermore, genetic mutations causing diseases in humans can easily be introduced to equivalent genes in zebrafish through, for instance, CRISPR-Cas9. This allows for high-throughput screens of potential medicines on a complete organism in which effects on all organs are easily observable. Of course, a zebrafish is not a human, so everything you find in zebrafish has to be validated on human tissue.

**What has surprised you the most while conducting your research?**

The specificity of several fungal compounds on zebrafish development is quite remarkable. When we first started this project, we did not really know what to expect. There are all these microorganisms in their own environment making all sorts of compounds for completely different purposes; for instance, to defend themselves or to communicate. You would not necessarily think that these compounds could act in specific pathways in vertebrates, but yet some turn out to have very specific targets and effects.

**Describe what you think is the most significant challenge impacting your research at this time and how will this be addressed over the next 10 years?**

The biggest challenge is translating the results of promising compounds on model organisms to use as medicine on humans. It is a long and an understandably elaborate process. A lot of screening on human tissue is still done in 2D cell culture on a selection of cell types. The emergence of 3D systems such as organoids has made testing on more complex human tissue easier. The next step, which is already in development, is combining all these different organoids in one system, to be able to test compounds on as many cell types at once as possible and thus reducing the gap from the lab to patient.

“A good scientist is determined by the way they design and perform experiments and conduct the scientific method, not necessarily by the outcome of these experiments.”

**Figure:**
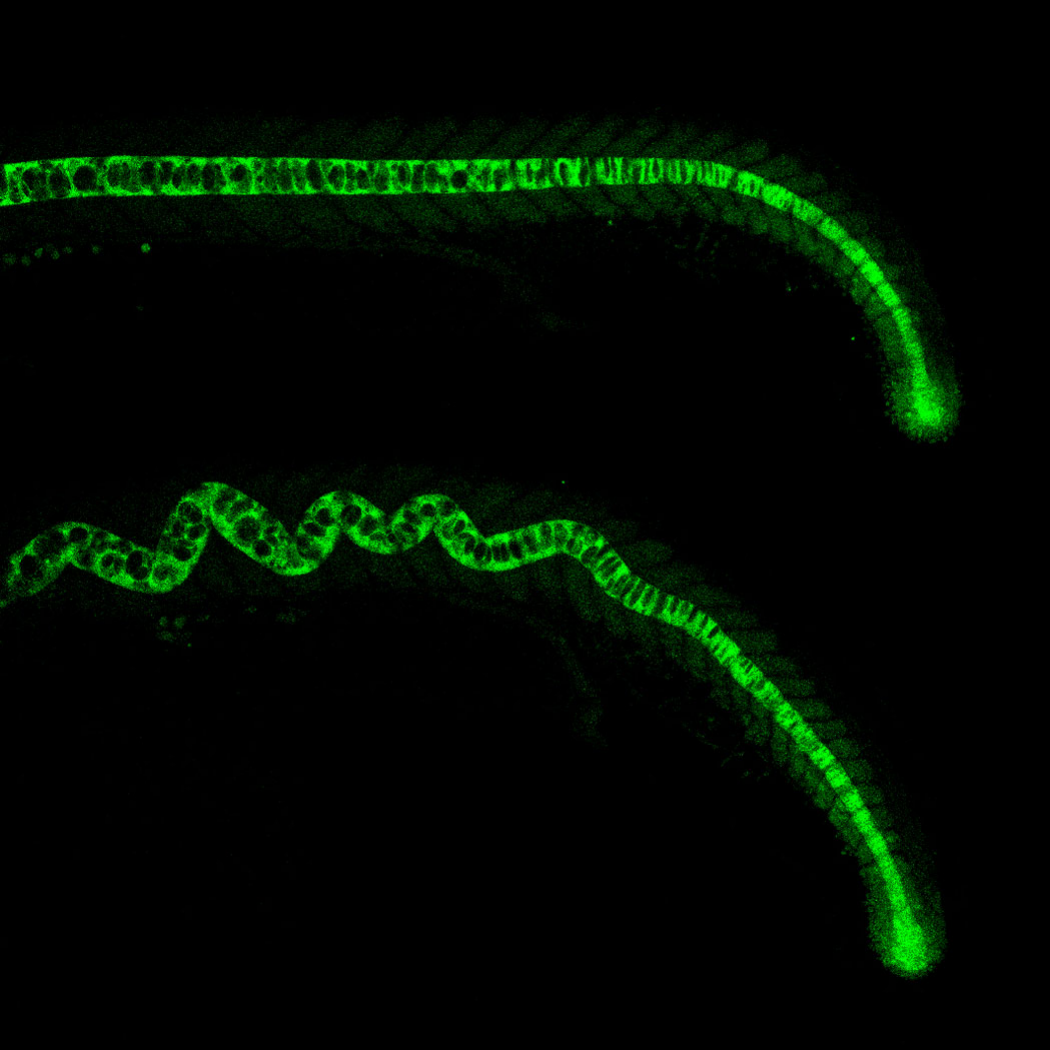
**Untreated transgenic *ntl*:*gfp* zebrafish embryo (above) versus an embryo treated with fungal compound fusaric acid (below), causing an undulating notochord.**

**What changes do you think could improve the professional lives of early-career scientists?**

Less pressure to publish (high). Young scientists often need to publish a certain number of (high-impact) papers in several years to get a degree, a grant or a chance to stay in science. This leads to working overtime, stressed scientists and rushed science, especially when a project is just not working and papers need to be produced. Ultimately, this goes at the cost of quality of science and life. Scientists should get the time and mentoring to perform proper science, regardless of whether this results in ten, two or zero papers. A good scientist is determined by the way they design and perform experiments and conduct the scientific method, not necessarily by the outcome of these experiments.

**What's next for you?**

For now, I am continuing to look for (new) compounds and understanding their effects. Thus far, we only investigated a small part of these fungi and phenotypes. There remains a lot to be found; we have only scratched the surface.
